# Temporal trend of accidents due to percutaneous exposure in a public hospital in Brazil, 2007-2019

**DOI:** 10.1590/0034-7167-2022-0046

**Published:** 2022-08-22

**Authors:** Renan Sallazar Ferreira Pereira, Cecília Angelita dos Santos, Adriano Marçal Pimenta

**Affiliations:** I Universidade Federal do Tocantins. Palmas, Tocantins, Brazil; II Hospital Municipal Dr. José de Carvalho Florence. São José dos Campos, São Paulo, Brazil; III Universidade Federal do Paraná. Curitiba, Paraná, Brazil

**Keywords:** Health Personnel, Accidents, Occupational, Needlestick Injuries, Hospitals, Interrupted Time Series Analysis., Personal de Salud, Accidentes de Trabajo, Lesiones por Pinchazo de Aguja, Hospitales, Estudios de Series Temporales., Pessoal de Saúde, Acidentes de Trabalho, Ferimentos Penetrantes Produzidos por Agulha, Hospitais, Estudos de Séries Temporais.

## Abstract

**Objectives:**

to analyze the temporal trend of accidents due to percutaneous exposure in a public hospital in Brazil, between 2007 and 2019, according to sociodemographic and professional characteristics.

**Methods:**

analysis of time series of accidents due to percutaneous exposure that occurred in health workers. Sociodemographic and professional variables, accident profile, post-accident behavior and accident incidence rates were evaluated. The Prais Winsten regression was used for trend analysis and calculation of the annual percentage change, with a significance level of 5%.

**Results:**

761 occupational accidents were recorded. There was a downward trend in the rate of percutaneous injuries among female workers (-0.012%; p=0.009), who had secondary education (-0.011%; p=0.035) and among all health professional categories (-0.010%; p =0.019). There was an increasing trend (0.018%; p= 0.050) among workers with ≥ 61 months of professional experience.

**Conclusions:**

the analysis showed a decreasing incidence of percutaneous accidents, which can be explained by multiple factors.

## RESUMO

**Objetivos:**

analisar a tendência temporal dos acidentes por exposição percutânea em um hospital público no Brasil, entre 2007 e 2019, segundo características sociodemográficas e profissionais.

**Métodos:**

análise de séries temporais dos acidentes por exposição percutânea ocorridos em trabalhadores de saúde. Foram avaliadas as variáveis sociodemográficas, profissionais, perfil dos acidentes, condutas pós-acidentes e as taxas de incidência dos acidentes. A regressão de Prais Winsten foi empregada para análise de tendência e cálculo da variação percentual anual, com nível de significância de 5%.

**Resultados:**

foram registrados 761 acidentes ocupacionais. Houve tendência decrescente da taxa de acidente percutâneo nos trabalhadores do sexo feminino (-0,012%; p=0,009), que possuíam ensino médio (-0,011%; p=0,035) e entre todas as categorias profissionais de saúde (-0,010%; p=0,019). Observou-se tendência crescente (0,018%; p= 0,050) entre trabalhadores com tempo ≥ 61 meses de experiência profissional.

**Conclusões:**

a análise evidenciou incidência decrescente de acidentes percutâneos, que pode ser explicada por múltiplos fatores.

## RESUMEN

**Objetivos:**

analizar tendencia temporal de accidentes por exposición percutánea en un hospital público brasileño, entre 2007 y 2019, segundo características sociodemográficas y profesionales.

**Métodos:**

análisis de series temporales de los accidentes por exposición percutánea ocurridos en trabajadores de salud. Fueron evaluadas las variables sociodemográficas, profesionales, perfil de los accidentes, conductas post-accidentes y tasas de incidencia de los accidentes. La regresión de Prais-Winsten fue empleada para análisis de tendencia y cálculo de la variación porcentual anual, con nivel de significación de 5%.

**Resultados:**

fueron registrados 761 accidentes ocupacionales. Hubo tendencia decreciente de la tasa de accidente percutáneo en trabajadores del sexo femenino (-0,012%; p=0,009), que poseían enseñanza media (-0,011%; p=0,035) y entre todas categorías profesionales de salud (-0,010%; p=0,019). Observado tendencia creciente (0,018%; p= 0,050) entre trabajadores con tiempo ≥ 61 meses de experiencia profesional.

**Conclusiones:**

el análisis evidenció incidencia decreciente de accidentes percutáneos, que puede ser explicada por múltiples factores.

## INTRODUCTION

There is a high worldwide prevalence of occupational accidents with exposure to potentially contaminated biological material (OAEBM), percutaneously (32.4%; 95% CI: 22.0 to 44.8) and in health workers (56.2 %; 95% CI: 47.1 to 64.9)^(1)^. Inadequacy of management strategies and lack of adherence to standard precautions are the main factors associated with high prevalence^(2)^.

The World Health Organization points out that, each year, about 2 million health workers are exposed to bloodborne pathogens (BBP) due to injuries caused by needles and other sharps (NS)^(3)^. Exposure caused by these types of materials subjects the worker to dozens of BBPs, such as hepatitis B (HBV), hepatitis C (HCV) and human immunodeficiency (HIV) viruses^(4)^.

In addition to the possibility of post-accident infection, percutaneous accidents can result in mental disorders, post-traumatic stress disorders, malaise due to post-exposure prophylaxis, absenteeism and, finally, the cost of treatment for the health service^(5-6)^. It is estimated that each accident case generates costs between 175 and 350 US dollars (USD) for health systems^(6)^.

The latest estimate made by the Centers for Disease Control and Prevention points to the annual occurrence of 385,000 cases of NS accidents in American hospitals, with an average of 1,000 cases per day. Within hospital institutions, the occurrence of accidents is associated with the professional category, time of professional experience, training status and working conditions^(7)^.

In Brazil, of the 53,524 accidents recorded in the hospital sector in 2017, 9,846 were due to contact with exposure to a communicable disease, a category that includes percutaneous OAEBMs^(8)^. While relevant, these numbers may be inaccurate. It is known that there is underreporting of statistics on accidents at work, restricting knowledge of the global magnitude of the problem and making it difficult to assess the effects of prevention measures^(9)^.

When considering the epidemiological significance of accidents with NS in health professionals in the hospital area, it is verified that research is carried out with the purpose of knowing the magnitude of accidents in these places^(1)^. However, most of these works are restricted to cross-sectional studies, with a short period of analysis of accident rates. In addition, there is the use of information from the Information System on Notifiable Diseases (SINAN), which has a high percentage of incomplete data^(10)^. In this regard, to date, there are few studies in the literature on the analysis of the historical series of percutaneous accidents and with a longitudinal follow-up of the incidence over ten years in hospitals.

## OBJECTIVES

To analyze the temporal trend of accidents due to percutaneous exposure in a public hospital in Brazil, between 2007 and 2019, according to sociodemographic and professional characteristics.

## METHODS

### Ethical aspects

The study was approved by the Ethics and Research Committee with Human Beings and complied with the norms of Resolution 466/12 of the National Health Council of the Ministry of Health.

### Study design, period, and location

This is a time series analysis of percutaneous OAEBMs that occurred in health workers at a public hospital, between 2007 and 2019. The writing of the present study was guided by the Strengthening the Reporting of Observational Studies in Epidemiology (STROBE) checklist^(11)^.

The study was developed in a large hospital, a reference for urgent and emergency care, which carries out research, teaching, and assistance activities. The institution is located in the municipality of São José dos Campos, state of São Paulo, (SP), Brazil. Among other features, since 2016, the hospital is accredited by the National Accreditation Organization (ONA).

### Population or sample; inclusion and exclusion criteria

The population consisted of all health workers who provided direct assistance to hospitalized individuals and who had an employment relationship with the hospital; and by students of the medical residency program. Those who performed exclusively administrative activities and those who were on leave/away from work were excluded.

### Study protocol

The workers’ information was extracted from the database of the Specialized Service in Safety Engineering and Occupational Medicine (SESMT), located on the hospital’s premises. The variables of interest were collected by a researcher, from February to August 2020, by completing a semi-structured form, prepared based on the literature^(12)^. The form contained the following information: sociodemographic data (gender, age and education); professional data (professional category and time in the institution); accident profile (place of occurrence of the accident, shift of occurrence, type of sharps, presence of a safety device, region of the body affected, circumstance of the accident, use of personal protective equipment - PPE); post-accident conduct data (notification of the work accident, issuance of the communication of the work accident, specialized medical evaluation, time of absence from work, serological status of the source and the professional, type of source, result of the Anti-HBs test, number of doses of hepatitis B vaccine received, indication of post-exposure prophylaxis - PPE for HIV, HBV, HCV, case evolution); number of accidents due to percutaneous exposure recorded; and number of workers exposed to the risk of accident due to percutaneous exposure.

Initially, the characterization of percutaneous accidents in the groups of health workers was carried out with a descriptive analysis through the calculation of absolute and relative frequencies. Then, the monthly incidence rates of percutaneous OAEBMs were calculated, according to sociodemographic data and professional category. The rate was obtained by dividing the monthly number of cases of “occupational accident due to percutaneous exposure” - based on the diagnosis of an occupational accident with exposure to biological material, Z20.9 in the 10th revision of the International Classification of Diseases (ICD-10) - by the number of health professionals exposed to the risk of OAEBM who provided direct care to hospitalized individuals in the month of occurrence, multiplying the result by 100. Finally, the average monthly rates were calculated for each calendar year.

### Analysis of results and statistics

The generalized linear regression of Prais-Winsten^(13-14)^ was used to analyze the temporal trend and calculate the annual percentage change in accident rates. This method is recommended for temporal trend studies, since the procedure considers serial autocorrelation^(13)^.

Regarding the evaluation of annual variations in rates obtained by the regression coefficients, all estimates indicated: (increasing) increase in the trend when the annual change in rates was positive and (decreasing) reduction in the trend when the annual change was negative.

All analyzes were performed using STATA^®^ software (version 14.0.) at a statistical significance level set at 5%.

## RESULTS

In the period from 2007 to 2019, 761 OAEBMs were recorded percutaneously among health professionals.

The highest proportion of records occurred with female workers (74.2%), aged between 30 and 39 years (42.2%), with high school education (53.7%). When considering the professional categories, higher records were observed among nursing technicians and assistants (53.9%), followed by the medical category (31.8%). Most (83.4%) had up to 60 months of professional experience at the hospital (Table 1).

**Table 1:** Absolute and relative distribution of occupational accidents with exposure to potentially contaminated biological material, percutaneously, that occurred in a public hospital, according to sociodemographic and professional data, São José dos Campos, São Paulo, Brazil, 2007-2019 (N = 761)

Variable	Total	
	n	%
Sex		
Male	196	25.8
Female	565	74.2
Age (years)		
18-29	286	37.6
30-39	321	42.2
40-49	112	14.7
≥ 50	42	5.5
Education (levels)		
High school	409	53.7
Higher education	352	46.3
Professional category		
Nurse	91	12.0
Technician and Nursing assistant	410	53.9
Physician	242	31.8
Other technical professionals^*^	8	1.1
Other higher-level professionals^**^	10	1.3
Time at the institution (months)		
≤ 60	635	83.4
≥ 61	126	16.6

* *Laboratory technician and assistant; pathology technician; radiology technician; dental assistant.*

* ^*^Biologist; biomedic; dental surgeon; physiotherapist.

**Table 2:** Absolute and relative distribution of occupational accidents with exposure to potentially contaminated biological material, percutaneously, that occurred in a public hospital, according to accident profile data, São José dos Campos, São Paulo, Brazil, 2007-2019 (N = 761)

Variable	Total	
	n	%
Location of accident (sector)		
ICUs	129	17.0
Surgery Center	217	28.5
Emergency / first aid	205	26.9
clinical-surgical ward	123	16.2
Others^*^	87	11.4
Shift in which the accident occurred (period)		
Morning day	253	33.3
Daytime afternoon	313	41.1
Nocturnal	195	25.6
Type of sharp material		
Needle with lumen	510	67.0
Suture needle	119	15.6
Scalpel blade	61	8.0
Surgical instruments	46	6.0
Others^†^	25	3.3
Presence of security device		
No	383	50.3
Yes	135	17.7
Non applicable	243	31.9
Affected body region		
Fingers	621	81.6
Hands	121	15.9
Others^‡^	19	2.5
Circumstance of the accident		
Improper sharps disposal	160	21.0
Adm. medication/vascular access/blood collection	322	42.3
Surgical/dental procedure	176	23.1
In material decontamination/organization/processing	43	5.7
Others^#^	60	7.9
Use of personal protective equipment		
Yes	663	87.1
No	98	12.9

* *Others: blood bank; PHD; outpatient clinic; NHE; pediatrics; CME; maternity; Clinical Laboratory. †Others: glass; scissors; lancet; bone fragment; razor blade; drain; chipped wood; glue bottle; pacemaker needle. ‡Others: leg; thigh; arm; forearm. #Others: needle recapping; withdrawal of points; debridement; trichotomy; assistance with movement needs; restriction; material preparation.*

Accidents occurred in several sectors of the public hospital, the most frequent being: operating room (28.5%), emergency/emergency room (26.9%) and intensive care units (17.0%). In these places, the highest proportion of accidents occurred in the afternoon (41.1%). The main type of NS involved in accidents was needle with lumen (67.0%), without safety device (50.3%). The most affected body region were the fingers (81.6%). Among the main tasks and/or circumstances that caused the accidents, the most important were medication administration, vascular access, blood sample collection (42.3%) and surgical/dental procedures (23.1%). It should be noted that most workers (87.1%) used PPE at the time of the accident (Table 1).

Regarding the conduct performed with the injured worker, the accident was notified by completing the SINAN form (CID Z20.9) and post-accident specialized medical evaluation in all 761 cases; and, consequently, the Work Accident Report (WAR) was issued in 98.2% of accidents. Among the injured, only 16.1% required time off work (data not shown).

To determine the post-exposure management, clinical and laboratory tests were performed both on the source patient and on the injured health professional (83.7%). Most of the serological test results (76.7%) of the source person/patient were negative for HIV, hepatitis B and C. However, a part had a positive result (5.9%) and/or indeterminate (1, 3%), thus indicating the need to introduce post-exposure prophylaxis (PEP) for HIV (4.5%), PEP for HBV (4.9%). In addition to the serological tests, a concern was the vaccination status against hepatitis B and the serology against the virus, which indicate vaccine efficacy: most of the victims had a record of three doses (91.7%) and antibodies (94.1%) (Table 3).

**Table 3:** Absolute and relative distribution of the proportion of occupational accidents with exposure to potentially contaminated biological material, percutaneously, that occurred in a public hospital, according to data on post-accident behavior, vaccination status and serological monitoring, São José dos Campos, São Paulo, Brazil, 2007-2019 (N = 761)

Variable	Total	
	n	%
Source and professional status^*^		
Only from the professional	124	16.3
From the source and the professional	637	83.7
Source type (serological result)		
Unknown	122	16.0
Negative (uninfected source patient)	584	76.7
Indeterminate (indeterminate - false positive/negative)	10	1.3
Positive for HIV/hepatitis B or C	45	5.9
Anti-HBs test result		
Responder† (reagent)	716	94.1
Non-responder^‡^	43	5.7
No results	2	0.3
Number of doses received of the hepatitis B vaccine		
No registration of doses	4	0.5
< 3 doses	14	1.8
Three doses (full schedule)	698	91.7
4-6 doses	45	5.9
Indication of post-exposure prophylaxis (PEP) for HIV		
Yes	34	4.5
No	727	95.5
Indication of PEP for HBV		
Yes	37	4.9
No	724	95.1
Conducts before the accident with exposure to HCV		
Yes (clinical laboratory follow-up)^#^	176	23.1
No	585	76.9
Evolution of the case		
Discharge without serological conversion	747	98.2
High with serological conversion	0	0.0
Abandonment of clinical laboratory follow-up	2	0.3
Unknown evolution^##^	12	1.6

* *Serological status based on hepatitis B virus surface antigen (HBsAg) detection test/examination, hepatitis B virus core antigen antibodies (anti-HBc), hepatitis C virus antibodies (anti-HCV) and antibodies against the human immunodeficiency virus (anti-HIV) antigen. †Responder: is defined as a person who has an adequate level of antibody to hepatitis B surface antigen (anti-HBs) ≥ 10 IU/L. ‡Non-responder/non-reactive: inadequate vaccination is defined as Anti-HBs < 10 IU/L. #Refers to the monitoring of workers exposed to the human immunodeficiency virus (HIV), hepatitis C virus (HCV) and hepatitis B virus (HBV). ##Unknown evolution: case in progress/forwarded to follow up in another institution.*

The conduct in the face of exposure to HCV led a part of the workers to specific clinical laboratory follow-up (23.1%). As for the evolution of the cases, the majority (98.2%) were discharged without serological conversion to the pathogens of blood exposure. There were few records of abandonment of clinical laboratory follow-up (0.3%) (Table 3).

**Figure 1:**
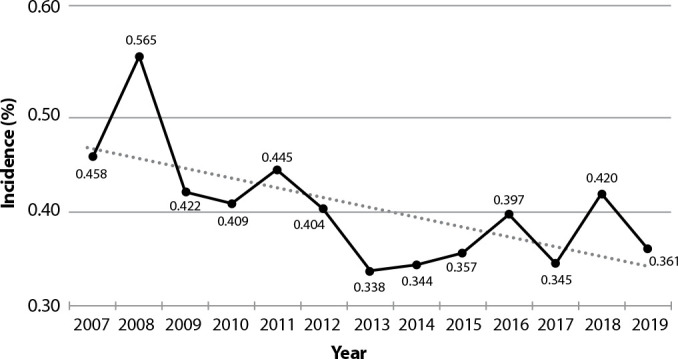
Temporal trend of the average monthly incidence rates of occupational accidents with exposure to potentially contaminated biological material, percutaneously, that occurred in a public hospital, according to all professional categories, São José dos Campos, São Paulo, Brazil, 2007-2019

Overall, a downward trend in accident rates was observed over the analyzed period (-0.010; p=0.019), with a peak incidence recorded in 2008 (approximately 0.6 accidents per 100 health workers/year) (Figure 1).

*Annual percentage change (-0.010; p=0.019), Waldman test of linear trend obtained by Prais-Winsten regression.*

Regarding the trend analysis of sociodemographic and professional data, from 2007 to 2019, there was an increase in the negative annual percentage change in percutaneous OAEBM rates among female workers (-0.012%; p=0.009) and those who they had only high school education (-0.011%; p=0.035). The increase in the positive annual percentage change for the category of workers with time equal to or greater than 61 months of service at the institution was at the threshold of statistical significance (0.018%; p=0.050) (Table 4).

**Table 4:** Distribution of the average monthly incidence rates of occupational accidents with exposure to potentially contaminated biological material, percutaneously, that occurred in a public hospital, according to professional category, São José dos Campos, São Paulo, Brazil, 2007-2019

Variable						%								Yearly change (%)	*p* value^*^
	2007	2008	2009	2010	2011	2012	2013	2014	2015	2016	2017	2018	2019		
Sex															
Male	0.4	0.6	0.2	0.5	0.4	0.4	0.2	0.3	0.2	0.4	0.3	0.4	0.3	-0.010	0.061
Female	0.5	0.6	0.5	0.4	0.5	0.4	0.4	0.4	0.4	0.4	0.4	0.4	0.4	-0.012	**0.009**
Age (you)															
18-29	0.9	0.8	0.8	0.7	0.8	0.8	0.7	0.7	0.6	1.0	0.7	0.9	1.0	0.008	0.377
30-39	0.4	0.5	0.4	0.4	0.5	0.4	0.3	0.3	0.4	0.4	0.4	0.5	0.4	-0.004	0.398
40-49	0.2	0.4	0.2	0.3	0.2	0.2	0.3	0.2	0.2	0.2	0.3	0.2	0.2	-0.007	0.083
≥ 50	0.3	0.5	0.2	0.2	0.2	0.1	0.0	0.1	0.2	0.0	0.0	0.3	0.2	-0.016	0.160
Education (levels)															
High school	0.4	0.6	0.5	0.4	0.4	0.4	0.3	0.4	0.4	0.4	0.4	0.3	0.3	-0.011	**0.035**
Higher education	0.5	0.6	0.3	0.4	0.5	0.5	0.4	0.3	0.3	0.4	0.3	0.5	0.4	-0.009	0.167
Professional category															
Nurse	0.9	1.1	0.7	0.2	0.5	0.4	0.5	0.4	0.3	0.2	0.2	0.7	0.5	-0.036	0.122
Technician and assistant of nursing	0.5	0.6	0.5	0.4	0.5	0.4	0.4	0.4	0.5	0.4	0.5	0.4	0.4	-0.009	0.096
Physician	0.3	0.5	0.3	0.5	0.5	0.5	0.3	0.4	0.2	0.5	0.3	0.5	0.4	-0.001	0.923
Other technical professionals^†^	0.2	0.0	0.6	0.0	0.0	0.0	0.0	0.0	0.0	0.3	0.0	0.0	0.1	-0.011	0.303
Other higher-level professionals^‡^	0.9	0.0	0.0	0.7	0.0	0.1	0.0	0.0	0.0	0.0	0.0	0.2	0.0	-0.031	0.062
Time at the institution (months)															
≤ 60	0.5	0.6	0.4	0.4	0.5	0.5	0.4	0.3	0.4	0.6	0.4	0.5	0.5	-0.001	0.895
≥ 61	0.0	0.0	0.1	0.2	0.2	0.2	0.2	0.3	0.2	0.1	0.2	0.3	0.2	0.018	**0.050**

* Linear trend Waldman test obtained by Prais-Winsten regression. Values in bold denote significant differences.

** Physiotherapist; biologist; biomedic; biochemical; dental surgeon; nutritionist; speech therapist; psychologist; laboratory technician; Occupational Therapist; laboratory assistant; dental assistant; pathology technician; radiology technician. †Laboratory assistant; dental assistant; pathology technician; radiology technician. ‡Physiotherapist; biologist; biomedic; biochemical; dental surgeon; nutritionist; speech therapist; psychologist; laboratory technician; Occupational Therapist.

## DISCUSSION

The time series of the incidence coefficient of OAEBMs percutaneously in health workers at the public hospital, from 2007 to 2019, was configured as a decreasing trend in general and among female professionals with high school education. It also showed a growing trend among workers with time equal to or greater than 61 months of professional experience in the hospital. The highest incidence rate was recorded in the second year of the historical series (2008). As a hypothesis, it is believed that the trends shown can be explained as a result of Brazilian legislation, biological, social, behavioral factors and the characteristics of the work process.

The peak incidence of percutaneous injuries observed in all professional categories in 2008 is lower than the incidence rates reported in Brazilian hospitals (7.5 cases per person/year) and higher than those found in Colombian hospitals (3.5 cases per person/year)^(15)^. This result is important for workers and the health institution under study. However, it is necessary to achieve better results, given the consequences of accidents. Strategies from countries with better rates of incidence of percutaneous accidents need to be analyzed regarding the feasibility for later implementation in Brazilian hospitals.

The downward trend in incidence observed among all health workers may not have been due to chance or underreporting. This downward trend may be associated with the hospital’s compliance with the Regulatory Norms of the Ministry of Labor, which advocate preventive control measures (PCM) and follow the guidelines of the National Occupational Health Policy, instituted in 2012^(16)^. These guidelines provided subsidies to improve worker health surveillance strategies.

The Regulatory Norm 32, aimed at protecting the safety and health of health service workers, implemented in 2005^(17)^, in some studies has shown to be correlated with the downward trend in the incidence of percutaneous accidents in certain Brazilian hospitals due to effect of PCMs^(15,18)^. International studies^(19-20)^ with similar legislation have also identified a downward trend in the rate of accidents due to percutaneous exposure among healthcare professionals in the hospital area, after the adoption of PCM. PCMs include: management of the use of engineering controls, controls of work practices, administrative controls, the use of PPE and training^(17,21)^. Such measures have been identified as the main factor responsible for the effect of reducing accidents within hospitals^(7,9,21-24)^.

It is also worth noting that the decline in rates occurred in a period when there was an improvement in accident records in Brazil^(20)^, and this fact may have acted as a reducer of the effect of PCMs. A study that evaluated the temporal trends of all OAEBM categories in Brazilian states, from 2010 to 2016, revealed a trend in the increase in accident rates among health workers, as a consequence of actions to combat underreporting and number of health professionals registered in each state^(25)^.

Education and training interventions have been widely used within hospitals to prevent NS injuries among health professionals^(26)^, especially in Brazil, after the implementation of NR 32^(17)^. Based on this information, education and training interventions may have had a reducing effect on the incidence of accidents in the female worker group and in the high school group. The training allows the worker to acquire a better safety culture, which makes it possible to identify risk situations, act safely and handle equipment correctly^(27-28)^.

Furthermore, one cannot rule out, as an explanatory hypothesis, the possibility of the influence of biological, social, behavioral characteristics and those linked to the specifics of the work process. Women take more care of their health than men, due to their physiological and social condition. The physiological demand of the female body accustoms women to adopt a health risk reduction behavior. This preventive behavior is stimulated by the concern about getting sick and, consequently, compromising the care of the children. The female gender pattern makes women more adherent to health interventions than when compared to men^(29)^.

Among health professionals, technicians who have only graduated from high school are the ones who most handle NS in invasive procedures. This characteristic confers a greater risk of percutaneous exposure^(2,7,30)^. As recommended by Brazilian legislation^(17)^, the replacement of PM by those that have safety devices combined with training for the correct use made mid-level professionals less exposed to the risk of OAEBM percutaneously during the work process. This may be an acceptable hypothesis to explain the downward trend among mid-level professionals. A study that analyzed the temporal trend in the rate of percutaneous injuries in a hospital during the progressive implantation of NS with safety devices to replace conventional NS observed a downward trend among professionals (nurses assistants) who have secondary education^(31)^.

The increasing temporal trend in the incidence of percutaneous accidents among workers with a time equal to or greater than 61 months of professional experience in the hospital can be explained by behavioral factors, such as difficulty in adhering to training^(32)^ and overconfidence to perform procedures invasive^(15,28)^. Despite the existence of PCMs and resources necessary to carry out safe work, it is often observed that more experienced health professionals put themselves at risk of an occupational accident, even in the possibility of contamination^(33-34)^.

### Study limitations

The study was carried out in a large hospital and with a seal of accreditation by the ONA provided in the years 2016 to 2019; therefore, it is necessary to be cautious with the external validity of our results. Multicenter studies can be conducted to verify whether the results of this work reflect national trends. Furthermore, although some hypotheses were pointed out about the factors responsible for the annual percentage change in the incidence rates of percutaneous accidents in the aforementioned hospital studied, future segmented regression analyzes for interrupted time series should be carried out, in order to attest to the effect of each intervention on health. On the other hand, as a positive point, this study used 11 time points (2007-2019), since, to identify statistically significant trends in time series, the use of seven or more points of analysis is recommended^(14)^.

### Contributions to the area of nursing, health, or public policy

Based on the historical series analysis proposed in this study, managers can restructure the actions to face percutaneous accidents foreseen in the occupational health service. In addition, the results are useful to expand the knowledge of researchers and nurses who work in the management area and seek alternative statistical methods to assess the temporal trend of accidents in health services.

## CONCLUSIONS

The present study analyzed the time series of the incidence coefficient of OAEBMs percutaneously in public hospital health workers, from 2007 to 2019; and showed a decreasing time trend in general and among female professionals and those with high school education. The growing trend in workers with 61 months or more of professional experience was on the threshold of statistical significance.

Health interventions, determined by public policies aimed at workers’ health, adopted in the hospital environment, and added to PCMs, may justify the observed downward trend. On the other hand, it is possible that behavioral factors were responsible for the growing secular trend among workers with longer service at the institution. Health intervention strategies and accident prevention involving sharps should be developed and implemented in the short term, in order to reduce the impacts of percutaneous accidents.

The results of this study provide information that makes it possible to plan and formulate preventive strategies to reduce the rate of percutaneous OAEBM in hospitals. It is suggested that managers observe the following intervention proposals: improve the organizational strategies adopted in existing programs in the institution that deal with risk reduction and establishment of measures to protect workers against occupational risks; carry out continuous training of workers on the correct use and disposal of NS; periodically test the theoretical knowledge and technical skills of professionals who use NS; encourage the development of a culture of safety and organization among health workers to identify risks and notify cases of accidents; restructure the organization of the work process in the sectors with accident records; regularly monitor the implementation of standard precautionary guidelines and workers’ adherence to them; standardize policies for the promotion and prevention of accidents and other health problems for workers in all sectors of the hospital environment; investing in engineering control in work environments and in improving working conditions; and maintain the quality of the accident information record for better analysis of indicators by the occupational health and safety service.
